# 2-Propyl-4*H*-thia­zolo[3,2-*a*][1,3,5]triazine-4-thione

**DOI:** 10.1107/S1600536808006752

**Published:** 2008-03-20

**Authors:** Uzma Yunus, Muhammad Kalim Tahir, Moazzam Hussain Bhatti, Wai-Yeung Wong

**Affiliations:** a Department of Chemistry, Allama Iqbal Open University, Islamabad, Pakistan; b Department of Chemistry, Hong Kong Baptist University, Waterloo Road, Kowloon Tong, Hong Kong

## Abstract

In the title compound, C_8_H_9_N_3_S_2_, the *n*-propyl chain is disordered over two orientations (site-occupancy ratio = 0.522:0.478) and is roughly perpendicular to the fused thia­zolotriazine system. The angle between the fused ring and the propyl chain is 83.6 (1)° [ 82.2 (1)° for the disordered chain]. The structure is stabilized by C—H⋯N hydrogen bonds.

## Related literature

For related literature, see: Jiang *et al.* (2007 ▶); Pauling *et al.* (1960 ▶); Yunus *et al.* (2007 ▶).
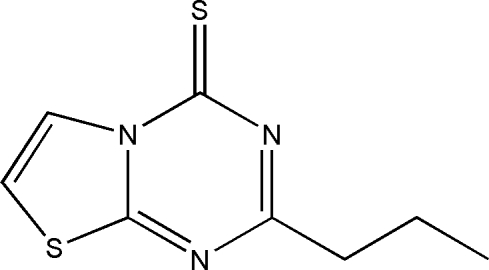


## Experimental

### 

#### Crystal data

C_8_H_9_N_3_S_2_
*M* *_r_* = 211.32
Monoclinic, 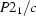

*a* = 9.3240 (7) Å
*b* = 14.9973 (11) Å
*c* = 6.8063 (5) Å
β = 95.505 (1)°
*V* = 947.37 (12) Å^3^
*Z* = 4
Mo *K*α radiation
μ = 0.52 mm^−1^
*T* = 173 (2) K
0.32 × 0.25 × 0.22 mm

#### Data collection

Bruker SMART CCD diffractometer
Absorption correction: multi-scan (*SADABS*; Bruker, 1999 ▶) *T*_min_ = 0.853, *T*_max_ = 0.895
5571 measured reflections
2254 independent reflections
2077 reflections with *I* > 2σ(*I*)
*R*_int_ = 0.016

#### Refinement

*R*[*F* ^2^ > 2σ(*F* ^2^)] = 0.024
*wR*(*F* ^2^) = 0.068
*S* = 1.05
2254 reflections
161 parameters
5 restraints
H-atom parameters constrained
Δρ_max_ = 0.32 e Å^−3^
Δρ_min_ = −0.28 e Å^−3^

### 

Data collection: *SMART* (Bruker, 1998 ▶); cell refinement: *SAINT* (Bruker, 1999 ▶); data reduction: *SAINT*; program(s) used to solve structure: *SHELXS97* (Sheldrick, 2008 ▶); program(s) used to refine structure: *SHELXL97* (Sheldrick, 2008 ▶); molecular graphics: *SHELXTL* (Sheldrick, 2008 ▶); software used to prepare material for publication: *SHELXTL*.

## Supplementary Material

Crystal structure: contains datablocks I, global. DOI: 10.1107/S1600536808006752/pk2086sup1.cif

Structure factors: contains datablocks I. DOI: 10.1107/S1600536808006752/pk2086Isup2.hkl

Additional supplementary materials:  crystallographic information; 3D view; checkCIF report

## Figures and Tables

**Table 1:** Hydrogen-bond geometry (Å, °)

*D*—H⋯*A*	*D*—H	H⋯*A*	*D*⋯*A*	*D*—H⋯*A*
C1—H1⋯N2^i^	0.95	2.38	3.3261 (16)	171

Symmetry code: (i) 
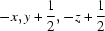
.

**Fig. 1.**
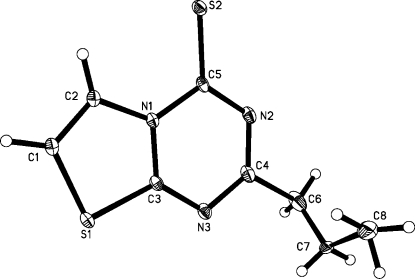
: A view of the molecular structure. Displacement ellipsoids are drawn at the 50% probability level.

## supplementary crystallographic information

### Comment

We have previously reported the crystal structure of 2-phenyl-4*H*-thiazolo[3,2,-a]- [1,3,5]triazine-4-thione with a phenyl group attached to the 1,3,5-triazine ring (Yunus *et al.*, 2007). The molecule was essentially planar. In contrast, in the title compound the pendant *n*-propyl group is almost perpendicular to the fused thiazolo[3,2,-a][1,3,5]triazine ring, which are themselves co-planar (maximum deviation from mean plane is 0.0437 (1) Å from atom C4). The *n*-propyl chain is disordered over two orientations with a site occupancy ratio of 0.522:0.478 (Jiang *et al.*, 2007). The CN bond distances of the 1,3,5-triazine ring are in the range 1.3191 (15) to 1.4093 (14) Å, in which N1—C5 bond length is slightly longer than that of N2—C5. These values are intermediate between those expected for single and double C—N bonds (1.47 and 1.27 Å, respectively). The C=S bond length of 1.6686 (12) Å is similar to that of the phenyl analog (Yunus *et al.*, 2007) but is slightly longer then the pure double bond distance (1.61 Å) (Pauling 1960). The bond angles and bond lengths in the thiazole ring are within the normal ranges. The crystal structure is stabilized by weak C—H···N hydrogen bonding interactions.

### Experimental

A mixture of ammonium thiocyanate (26 mmol) and butyryl chloride (26 mmol) in dry acetone (60 ml) was stirred for 30 min. Then 2-aminothiazole (26 mmol) was added and the reaction mixture was refluxed for 2 h. After cooling, the reaction mixture was poured into acidified cold water. The resulting yellow solid was filtered and washed with cold acetone. Single crystals of the title compound suitable for single-crystal *x*-ray analysis were obtained by recrystallization of the yellow solid from acetonitrile.

### Refinement

H atoms were found in difference Fourier maps and subsequently placed in idealized positions with constrained C—H distances of 0.98 Å (RCH_3_), 0.99 Å (*R*_2_CH_2_) and 0.95 Å (C_Ar_H) with *U*_iso_(H) values set to either 1.5*U*_eq_ (RCH_3_) or 1.2*U*_eq_ of the attached C atom.

### Crystal data

**Table:**

C_8_H_9_N_3_S_2_	*F*_000_ = 440
*M**_r_* = 211.32	*D*_x_ = 1.481 Mg m^−^^3^
Monoclinic, *P*2_1_/*c*	Mo *K*α radiation λ = 0.71073 Å
Hall symbol: -P 2ybc	Cell parameters from 5571 reflections
*a* = 9.3240 (7) Å	θ = 2.6–28.3º
*b* = 14.9973 (11) Å	µ = 0.52 mm^−^^1^
*c* = 6.8063 (5) Å	*T* = 173 (2) K
β = 95.505 (1)º	Block, pale yellow
*V* = 947.37 (12) Å^3^	0.32 × 0.25 × 0.22 mm
*Z* = 4	

### Data collection

**Table:**

Bruker SMART CCD diffractometer	2254 independent reflections
Radiation source: fine-focus sealed tube	2077 reflections with *I* > 2σ(*I*)
Monochromator: graphite	*R*_int_ = 0.016
*T* = 173(2) K	θ_max_ = 28.3º
ω and φ scans	θ_min_ = 2.6º
Absorption correction: multi-scan(SADABS; Bruker, 1999)	*h* = −12→12
*T*_min_ = 0.853, *T*_max_ = 0.895	*k* = −19→14
5571 measured reflections	*l* = −9→9

### Refinement

**Table:**

Refinement on *F*^2^	Secondary atom site location: difference Fourier map
Least-squares matrix: full	Hydrogen site location: inferred from neighbouring sites
*R*[*F*^2^ > 2σ(*F*^2^)] = 0.024	H-atom parameters constrained
*wR*(*F*^2^) = 0.068	*w* = 1/[σ^2^(*F*_o_^2^) + (0.0392*P*)^2^ + 0.2601*P*] where *P* = (*F*_o_^2^ + 2*F*_c_^2^)/3
*S* = 1.05	(Δ/σ)_max_ = 0.001
2254 reflections	Δρ_max_ = 0.32 e Å^−^^3^
161 parameters	Δρ_min_ = −0.28 e Å^−^^3^
5 restraints	Extinction correction: SHELXL97 (Sheldrick, 2008), Fc^*^=kFc[1+0.001xFc^2^λ^3^/sin(2θ)]^-1/4^
Primary atom site location: structure-invariant direct methods	Extinction coefficient: 0.0066 (12)

### Special details

**Table:**

Geometry. All e.s.d.'s (except the e.s.d. in the dihedral angle between two l.s. planes) are estimated using the full covariance matrix. The cell e.s.d.'s are taken into account individually in the estimation of e.s.d.'s in distances, angles and torsion angles; correlations between e.s.d.'s in cell parameters are only used when they are defined by crystal symmetry. An approximate (isotropic) treatment of cell e.s.d.'s is used for estimating e.s.d.'s involving l.s. planes.
Refinement. Refinement of *F*^2^ against ALL reflections. The weighted *R*-factor *wR* and goodness of fit *S* are based on *F*^2^, conventional *R*-factors *R* are based on *F*, with *F* set to zero for negative *F*^2^. The threshold expression of *F*^2^ > 2σ(*F*^2^) is used only for calculating *R*-factors(gt) *etc*. and is not relevant to the choice of reflections for refinement. *R*-factors based on *F*^2^ are statistically about twice as large as those based on *F*, and *R*- factors based on ALL data will be even larger.

### Fractional atomic coordinates and isotropic or equivalent isotropic displacement parameters (Å^2^)

**Table:**

	*x*	*y*	*z*	*U*_iso_*/*U*_eq_	Occ. (<1)
S1	0.20093 (3)	0.710163 (18)	0.27470 (4)	0.02095 (10)	
S2	−0.15462 (3)	0.443002 (19)	0.21702 (4)	0.02265 (10)	
N1	0.04853 (10)	0.56884 (6)	0.25767 (13)	0.01739 (19)	
N2	0.12868 (11)	0.42185 (7)	0.29852 (15)	0.0235 (2)	
N3	0.29929 (11)	0.54164 (7)	0.31895 (15)	0.0231 (2)	
C1	0.01557 (13)	0.71890 (8)	0.23196 (17)	0.0224 (2)	
H1	−0.0344	0.7739	0.2143	0.027*	
C2	−0.04951 (12)	0.63902 (8)	0.22664 (17)	0.0211 (2)	
H2	−0.1508	0.6311	0.2043	0.025*	
C3	0.18861 (12)	0.59613 (7)	0.28536 (15)	0.0185 (2)	
C5	0.01588 (12)	0.47711 (7)	0.25939 (15)	0.0187 (2)	
C4	0.26174 (14)	0.45502 (8)	0.32834 (18)	0.0259 (3)	0.522 (4)
C6	0.3713 (6)	0.3883 (4)	0.4050 (7)	0.0221 (9)	0.522 (4)
H6A	0.3288	0.3278	0.4003	0.029 (8)*	0.522 (4)
H6B	0.4062	0.4022	0.5436	0.025 (7)*	0.522 (4)
C7	0.4958 (2)	0.39267 (16)	0.2751 (4)	0.0248 (6)	0.522 (4)
H7A	0.5357	0.4539	0.2799	0.021 (7)*	0.522 (4)
H7B	0.5730	0.3517	0.3292	0.036 (8)*	0.522 (4)
C8	0.4516 (9)	0.3679 (6)	0.0614 (6)	0.0357 (13)	0.522 (4)
H8A	0.5357	0.3714	−0.0143	0.030 (8)*	0.522 (4)
H8B	0.3773	0.4093	0.0055	0.045 (10)*	0.522 (4)
H8C	0.4134	0.3070	0.0552	0.061 (12)*	0.522 (4)
C4A	0.26174 (14)	0.45502 (8)	0.32834 (18)	0.0259 (3)	0.478 (4)
C6A	0.3938 (7)	0.3911 (4)	0.3487 (8)	0.0255 (12)	0.478 (4)
H6A1	0.4827	0.4276	0.3621	0.047 (11)*	0.478 (4)
H6A2	0.3913	0.3565	0.4722	0.038 (11)*	0.478 (4)
C7A	0.4034 (3)	0.32554 (16)	0.1781 (4)	0.0247 (7)	0.478 (4)
H7A1	0.4736	0.2781	0.2199	0.036 (9)*	0.478 (4)
H7A2	0.3083	0.2970	0.1461	0.037 (9)*	0.478 (4)
C8A	0.4491 (8)	0.3709 (6)	−0.0056 (8)	0.0320 (11)	0.478 (4)
H8A1	0.4583	0.3263	−0.1087	0.064 (14)*	0.478 (4)
H8A2	0.5420	0.4007	0.0263	0.061 (13)*	0.478 (4)
H8A3	0.3764	0.4152	−0.0528	0.030 (9)*	0.478 (4)

### Atomic displacement parameters (Å^2^)

**Table:**

	*U*^11^	*U*^22^	*U*^33^	*U*^12^	*U*^13^	*U*^23^
S1	0.02618 (16)	0.01417 (15)	0.02233 (16)	−0.00294 (10)	0.00150 (11)	0.00001 (9)
S2	0.02469 (16)	0.01845 (15)	0.02542 (16)	−0.00432 (10)	0.00559 (11)	−0.00188 (10)
N1	0.0225 (4)	0.0139 (4)	0.0155 (4)	−0.0004 (3)	0.0008 (3)	0.0001 (3)
N2	0.0307 (5)	0.0151 (4)	0.0231 (5)	0.0010 (4)	−0.0059 (4)	−0.0003 (4)
N3	0.0254 (5)	0.0183 (5)	0.0243 (5)	0.0009 (4)	−0.0051 (4)	−0.0020 (4)
C1	0.0273 (6)	0.0166 (5)	0.0236 (6)	0.0020 (4)	0.0037 (4)	−0.0001 (4)
C2	0.0235 (5)	0.0172 (5)	0.0226 (5)	0.0024 (4)	0.0029 (4)	0.0003 (4)
C3	0.0247 (5)	0.0157 (5)	0.0147 (5)	−0.0022 (4)	0.0000 (4)	−0.0010 (4)
C5	0.0280 (5)	0.0148 (5)	0.0130 (5)	−0.0015 (4)	0.0010 (4)	−0.0015 (4)
C4	0.0301 (6)	0.0183 (5)	0.0266 (6)	0.0025 (4)	−0.0106 (5)	−0.0023 (4)
C6	0.0217 (16)	0.0211 (14)	0.023 (2)	0.0022 (11)	−0.0009 (16)	0.0060 (18)
C7	0.0159 (11)	0.0253 (12)	0.0332 (13)	0.0029 (8)	0.0023 (9)	−0.0008 (9)
C8	0.0347 (17)	0.039 (2)	0.034 (3)	0.0051 (15)	0.006 (3)	−0.009 (3)
C4A	0.0301 (6)	0.0183 (5)	0.0266 (6)	0.0025 (4)	−0.0106 (5)	−0.0023 (4)
C6A	0.032 (3)	0.0196 (16)	0.023 (3)	0.0051 (16)	−0.0085 (19)	0.0019 (19)
C7A	0.0224 (11)	0.0180 (12)	0.0337 (15)	0.0042 (9)	0.0030 (10)	0.0003 (10)
C8A	0.0268 (16)	0.0325 (18)	0.038 (3)	−0.0014 (13)	0.009 (3)	0.004 (3)

### Geometric parameters (Å, °)

**Table:**

S1—C3	1.7161 (11)	C6—H6B	0.9900
S1—C1	1.7299 (13)	C7—C8	1.519 (5)
S2—C5	1.6686 (12)	C7—H7A	0.9900
N1—C3	1.3644 (14)	C7—H7B	0.9900
N1—C2	1.3967 (14)	C8—H8A	0.9800
N1—C5	1.4093 (14)	C8—H8B	0.9800
N2—C4	1.3340 (16)	C8—H8C	0.9800
N2—C5	1.3455 (15)	C6A—C7A	1.531 (6)
N3—C3	1.3191 (15)	C6A—H6A1	0.9900
N3—C4	1.3485 (15)	C6A—H6A2	0.9900
C1—C2	1.3418 (17)	C7A—C8A	1.520 (6)
C1—H1	0.9500	C7A—H7A1	0.9900
C2—H2	0.9500	C7A—H7A2	0.9900
C4—C6	1.488 (6)	C8A—H8A1	0.9800
C6—C7	1.526 (5)	C8A—H8A2	0.9800
C6—H6A	0.9900	C8A—H8A3	0.9800
			
C3—S1—C1	90.73 (6)	C8—C7—C6	113.1 (4)
C3—N1—C2	113.50 (9)	C8—C7—H7A	109.0
C3—N1—C5	119.76 (9)	C6—C7—H7A	109.0
C2—N1—C5	126.73 (10)	C8—C7—H7B	109.0
C4—N2—C5	119.89 (10)	C6—C7—H7B	109.0
C3—N3—C4	113.74 (10)	H7A—C7—H7B	107.8
C2—C1—S1	112.26 (9)	C7—C8—H8A	109.5
C2—C1—H1	123.9	C7—C8—H8B	109.5
S1—C1—H1	123.9	H8A—C8—H8B	109.5
C1—C2—N1	112.39 (10)	C7—C8—H8C	109.5
C1—C2—H2	123.8	H8A—C8—H8C	109.5
N1—C2—H2	123.8	H8B—C8—H8C	109.5
N3—C3—N1	124.11 (10)	C7A—C6A—H6A1	108.4
N3—C3—S1	124.77 (9)	C7A—C6A—H6A2	108.4
N1—C3—S1	111.11 (8)	H6A1—C6A—H6A2	107.5
N2—C5—N1	115.95 (10)	C8A—C7A—C6A	112.2 (4)
N2—C5—S2	123.99 (9)	C8A—C7A—H7A1	109.2
N1—C5—S2	120.05 (8)	C6A—C7A—H7A1	109.2
N2—C4—N3	126.44 (11)	C8A—C7A—H7A2	109.2
N2—C4—C6	113.6 (3)	C6A—C7A—H7A2	109.2
N3—C4—C6	119.4 (3)	H7A1—C7A—H7A2	107.9
C4—C6—C7	107.7 (3)	C7A—C8A—H8A1	109.5
C4—C6—H6A	110.2	C7A—C8A—H8A2	109.5
C7—C6—H6A	110.2	H8A1—C8A—H8A2	109.5
C4—C6—H6B	110.2	C7A—C8A—H8A3	109.5
C7—C6—H6B	110.2	H8A1—C8A—H8A3	109.5
H6A—C6—H6B	108.5	H8A2—C8A—H8A3	109.5
			
C3—S1—C1—C2	−0.03 (9)	C4—N2—C5—S2	179.23 (9)
S1—C1—C2—N1	0.23 (13)	C3—N1—C5—N2	3.03 (15)
C3—N1—C2—C1	−0.38 (14)	C2—N1—C5—N2	−178.04 (10)
C5—N1—C2—C1	−179.36 (10)	C3—N1—C5—S2	−177.64 (8)
C4—N3—C3—N1	−1.45 (16)	C2—N1—C5—S2	1.28 (15)
C4—N3—C3—S1	177.43 (9)	C5—N2—C4—N3	−1.78 (19)
C2—N1—C3—N3	179.37 (10)	C5—N2—C4—C6	169.6 (2)
C5—N1—C3—N3	−1.58 (16)	C3—N3—C4—N2	3.24 (19)
C2—N1—C3—S1	0.35 (11)	C3—N3—C4—C6	−167.7 (2)
C5—N1—C3—S1	179.41 (7)	N2—C4—C6—C7	129.4 (3)
C1—S1—C3—N3	−179.19 (10)	N3—C4—C6—C7	−58.6 (4)
C1—S1—C3—N1	−0.19 (8)	C4—C6—C7—C8	−62.5 (5)
C4—N2—C5—N1	−1.48 (16)		

### Hydrogen-bond geometry (Å, °)

**Table:**

*D*—H···*A*	*D*—H	H···*A*	*D*···*A*	*D*—H···*A*
C1—H1···N2^i^	0.95	2.38	3.3261 (16)	171

Symmetry codes: (i) −*x*, *y*+1/2, −*z*+1/2.
